# Demineralization, Collagen Modification and Remineralization Degree of Human Dentin after EDTA and Citric Acid Treatments

**DOI:** 10.3390/ma12010025

**Published:** 2018-12-21

**Authors:** Maria Giovanna Gandolfi, Paola Taddei, Anna Pondrelli, Fausto Zamparini, Carlo Prati, Gianrico Spagnuolo

**Affiliations:** 1 Laboratory of Biomaterials and Oral Pathology, Dental School, Department of Biomedical and NeuroMotor Sciences, University of Bologna, 40126 Bologna, Italy; mgiovanna.gandolfi@unibo.it (M.G.G.); anna.pondrelli@studio.unibo.it (A.P.); fausto.zamparini2@unibo.it (F.Z.); 2 Biochemistry Unit, Department of Biomedical and NeuroMotor Sciences, University of Bologna, 40126 Bologna, Italy; paola.taddei@unibo.it; 3 Endodontic Clinical Section, Dental School, Department of Biomedical and NeuroMotor Sciences, University of Bologna, 40126 Bologna, Italy; 4 Department of Neuroscience and Reproductive and Odontostomatological Sciences, University of Napoli “Federico II”, 80131 Napoli, Italy; gianrico.spagnuolo@gmail.com; 5 Institute of Dentistry, I. M. Sechenov First Moscow State Medical University, 119146 Moscow, Russia

**Keywords:** EDTA, citric acid, collagen modification, mineralization degree, chelating agents, Ca/P atomic ratio, Ca/N atomic ratio, P/N atomic ratio

## Abstract

The aim of the study was to investigate the effects of several decalcifying agents used as irrigant solutions in endodontic treatment on collagen and mineral components of dentin. Coronal dentin discs from five caries-free human third molars with a smear layer were treated for one minute with a chelating solution (1% Ethylenediaminetetraacetic acid (EDTA), 10% EDTA, 17% EDTA, 10% citric acid). Mineralization degree (Ca/N and P/N atomic ratios, IR *I*_apatite_/*I*_amide II_ and *I*_1410(carbonate)_/*I*_554(phosphate)_ spectroscopic ratios) and possible collagen rearrangements (collagen infrared (IR) amide II e III shifts) were evaluated by environmental scanning electron microscopy (ESEM)/energy dispersive X-ray spectroscopy (EDX) and IR spectroscopy before and after treatment (T0) and after ageing (T24h and T2m) in simulated body fluid (SBF). At T0, analysis showed that the highest demineralizing effect was achieved using a 10% citric acid solution and 10% EDTA, while the smallest effect was observed when using 17% EDTA. No significant collagen modifications were detected upon treatment with 1% EDTA, while subtle changes were observed after the other treatments. At T24h or T2m, analyses showed the highest remineralization values for 1% EDTA and the lowest for 10% citric acid, mainly at T2m. The samples treated with 17% EDTA showed slight collagen rearrangements upon remineralization. In conclusion, the highest demineralizing effect was observed for 10% EDTA and 10% citric acid. Collagen rearrangement was found for all the treatments except for 1% EDTA. The highest remineralization capability in SBF values was recorded for 1% EDTA and the lowest for 10% citric acid. A slight collagen rearrangement upon remineralization was still present in 17% EDTA-treated samples. Clinical use as a chelating agent in the endodontic therapy of citric acid and concentrated EDTA solutions should be reconsidered.

## 1. Introduction

Dentine is a complex tissue which contains collagen and other proteins, apatite as the mineral phase, and water. Dentine treatments with manual or NiTi rotary root canal instruments generate a smear layer [1,2,3,4], an amorphous irregular thin layer composed of both organic and inorganic components. This smear layer may be attached to the dentinal surface and forced into the dentinal tubules, forming intratubular plugs [5,6]. In endodontics, the smear layer covers the root canal walls and occludes the orifices of the dentinal tubules [1,7,8] preventing the complete sterilization of the canals and an appropriate seal. In order to have a complete removal of the smear layer, chelating agents are routinely used in endodontics.

Ethylenediaminetetraacetic acid (EDTA) and citric acid solutions are the most common chelating agents currently used for root canal treatment and are able to demineralize dentine by combining with calcium ions of the dentine structure [9]. EDTA is considered the most effective chelating agent in endodontic therapy, showing the ability to very effectively remove the inorganic component, especially in the coronal and middle third of the canal [9,10,11]. Similarly, citric acid solutions—at concentrations ranging from 1% to 50%—are used to remove both the superficial smear layer and intratubular smear plugs [12,13,14,15,16].

Despite the wide clinical use of EDTA and citric acid solutions, no information is present regarding their activity on both organic and inorganic components. In particular, collagen rearrangement/modifications produced by EDTA and citric acid solutions have been never investigated. Clinically, prolonged activity of residual chelating agents on the inorganic dentine structure may lead to apical leakage [17,18] and reduced tooth microhardness, finally resulting in endodontic failures and root fractures.

Different techniques allow for analysis of the samples in a non-destructive way and without invasive measurements, including Fourier transform infrared spectroscopy (FTIR) with attenuated total reflectance (ATR) [19,20,21] and environmental scanning electron microscopy (ESEM) with energy dispersive X-ray spectrometry (EDX) [22,23]. Together, these techniques may be useful for analyzing the changes in the degree of dentine mineralization and the collagen modifications after chemical treatments.

In complex biological systems, such as dentine, the IR spectrum is the sum of the contributions gathered mainly from collagen and apatite phases [23]. The intensities of IR absorption bands provide quantitative information about the sample contents, depending on the nature of the molecular bonds, their structure, and their environment [22]. Previous investigations have used IR spectroscopy to study dentine collagen mineralization [22,24,25,26] and useful spectroscopic markers have been identified to determine the changes in the apatite/collagen ratio [22,24].

ESEM enables the examination of soft, hydrated, unfixed, and uncoated surfaces, i.e., bulk biological tissues in their “natural” state, providing a great advantage for the studies in the biological field [27,28]. X-ray microprobe and EDX mapping allow for the identification of different mineralizing zones and the mapping of the demarcation between the mineralizing and the mineralized areas [20,22,23]. The evaluation of Ca/N and P/N ratios proved to be a useful method to evaluate the degree of mineralization of the organic matrix of dentine [20,22,23].

The present study was aimed at assessing the dentine de-remineralization process and the collagen rearrangement after treatment with different chelating acids (EDTA solutions and citric acid solution) and after immersion in simulated body fluid (SBF) for 24 h and two months. In particular, the Ca/N and P/N ratios calculated by EDX and IR spectroscopic markers were used to evaluate the degree of mineralization of the organic matrix of dentine.

## 2. Materials and Methods

### 2.1. Dentine Preparation and Treatment

Five dentine discs were prepared from the middle third of coronal human caries-free molars extracted for surgical reasons from healthy patients. The root and the occlusal enamel were removed from each tooth to obtain 2.0–0.5 mm-thick crown segments. The removal of pulpal tissue was performed with small forceps, taking care not to touch the predentine surface and the inner part of the pulpal chamber. After preparation of the dentine discs, each one was divided into four slices; two for the EDX analyses and two for the attenuated total reflectance (ATR)–Fourier transform infrared (FTIR) analyses. One slice was analyzed after the treatments and after 24 months, while the other slice was prepared and stored for two months. The upper dentine surface of each slice was sanded with wet 600-grit SiC abrasive paper for 30 s to create a standard flat dentine surface covered by a standardized smear layer (SL) [29,30]. Each dentine slice surface (*n* = 4 for each tooth) was then treated with 1 mL of one of the different chelating agents, namely EDTA (1%, 10%, 17%) or citric acid 10% (positive control group) or with distilled water (negative control group) for 1 min, see Table 1. The pH of each tested solution has been measured using a selective temperature-compensated electrode (Sen Tix Sur WTW, Weilheim, Germany) connected to a multi-parameter laboratory meter (InoLab 750 WTW, Weilheim, Germany) previously calibrated with standard solutions.

Each dentine slice was then rinsed with distilled water and soaked in SBF, whose composition was Ca^++^ 1.27 mM, Cl^−^ 144.7 mM, K^+^ 5.8 mM, Na^+^ 141.6 mM, Mg^++^ 0.81 mM, HCO_3_^−^ 4.17 mM, SO_4_^2−^ 0.81 mM, H_2_PO_4_^−^ 0.44 mM, and HPO_4_^2−^ 0.336 mM. At each experimental time, i.e., after the SL formation, after chelating agent application, after 24 h, and after two months of aging in SBF, the samples were tested for mineralization degree by EDX and ATR-FTIR and for surface morphology by ESEM. At each evaluation time, three measurements by both ESEM/EDX and ATR-FTIR were made on each dentine slice.

### 2.2. ESEM/EDX Analysis

At each step of the study, the dentine surfaces were examined using an environmental scanning electron microscope (ESEM, Zeiss EVO 50; Carl Zeiss, Oberkochen, Germany) connected to a secondary electron detector for energy dispersive X-ray analysis (EDX; Oxford INCA 350 EDS, Abingdon, UK) using computer-controlled software (Inca Energy Version 18). The uncoated surfaces were examined at low vacuum (100 Pascal), 20 kV accelerating voltage, 8.5 mm working distance, 0.5 wt % detection level, 133 eV resolution, 100 microseconds amplification time, measuring time: 600 s for element mapping and 60 s for spectra. EDX microchemical analysis with a ZAF correction method was carried out (*n* = 3 per sample) at random in full frame to analyze entire areas of approximately 50 × 50 µm to evaluate the relative element content (weight % and atomic %). The Ca/N and P/N ratios were calculated from the data to evaluate the degree of mineralization of the dentine surfaces; the Ca/P ratio was calculated to characterize the mineral phase [23].

### 2.3. FT-IR Analysis

The same dentine samples that were analyzed by EDX were submitted for ATR-IR surface analyses by a Bruker Alpha (Bruker Optik GmbH, Ettlingen, Germany) Fourier-Transform FT-IR spectrometer in ATR mode with a diamond inner reflection element (IRE). The *I*_apatite_/*I*_amide II_ and *I*_1410(carbonate)_/*I*_554(phosphate)_ absorbance ratios were calculated as peak heights by using the Jasco Spectra analysis software (Jasco Inc., Easton, MD, USA), version 1.53.03. At least three spectra were recorded for each sample at each step of the study. Average spectra were shown; *I*_apatite_/*I*_amide II_ and *I*_1410(carbonate)_/*I*_554(phosphate)_ values were reported as mean values ± standard deviation.

The reduction of the *I*_apatite_/*I*_amide II_ ratio indicated the occurrence of a demineralization, with the weakening of the apatite bands compared to those of collagen while increasing the ratio indicated a remineralization. The changes in the *I*_1410(carbonate)_/*I*_554(phosphate)_ ratio assessed the carbonate content of the B-type carbonated apatite. The shifts of the collagen bands (Amide II e III) allowed for evaluation of the collagen rearrangements. Under the used experimental conditions, the penetration into the sample thickness was about 2 µm.

### 2.4. Statistical Analysis

The data (expressed as mean ± standard deviation) were analyzed using one-way analysis of variance (ANOVA) for repeated measurements followed by a repeated measures (RM) Student–Newman–Keuls test among evaluation times and two-way ANOVA followed by an RM Student–Newman–Keuls test among solutions. The statistical significance was set at *p* < 0.05.

## 3. Results

**Figure 1:**
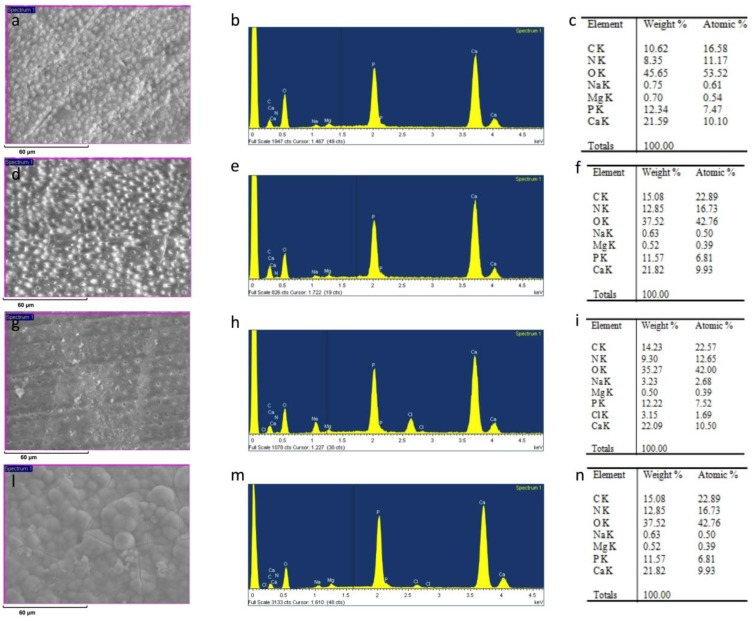
ESEM image, EDX spectra, and table of elements recorded on the dentin surface after sanding with abrasive paper (**a**,**b**,**c**), after treatment with a 1% EDTA solution (**d**,**e**,**f**), and after immersion in simulated body fluid (SBF) for 24 h (**g**,**h**,**i**) and two months (**l**,**m**,**n**).

**Figure 2:**
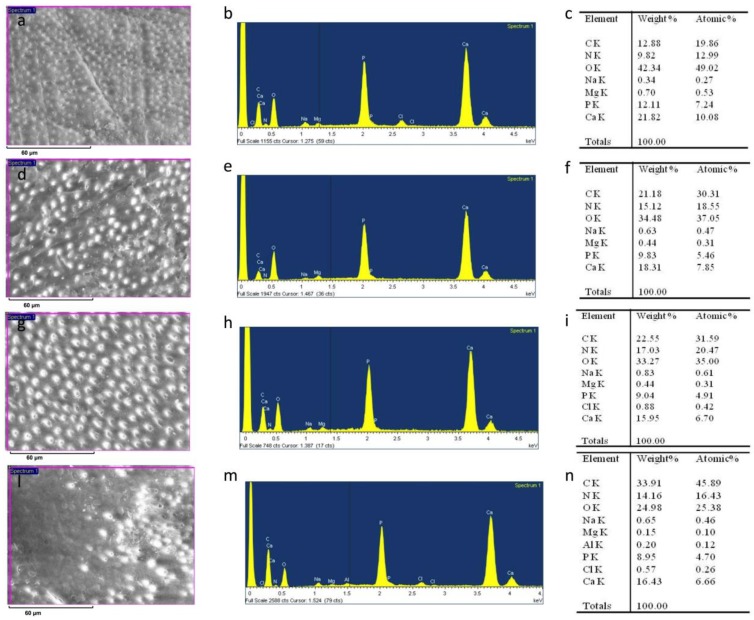
ESEM image, EDX spectra, and table of elements recorded on the dentin surface after sanding with abrasive paper (**a**,**b**,**c**), after treatment with a 10% EDTA (Tubuliclean 10%) (**d**,**e**,**f**), and after immersion in SBF for 24 h (**g**,**h**,**i**) and two months (**l**,**m**,**n**).

**Figure 3:**
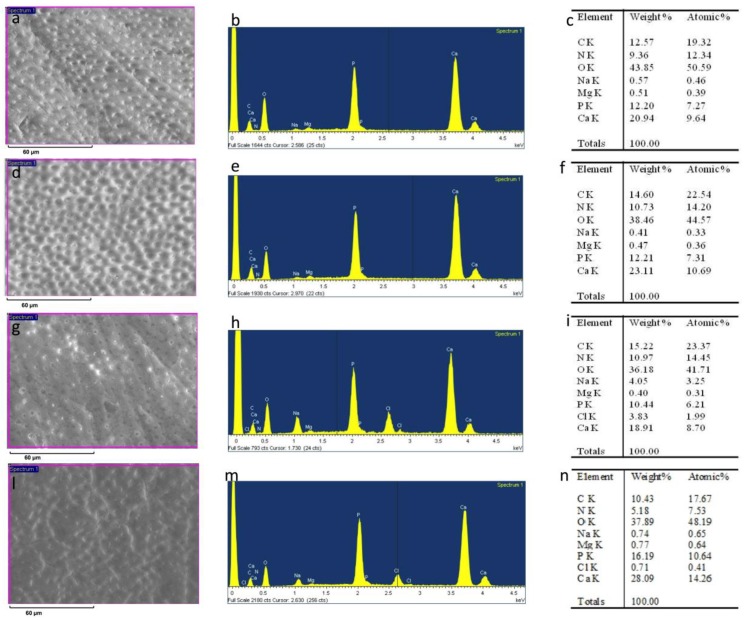
ESEM image, EDX spectra, and table of elements recorded on the dentin surface after sanding with abrasive paper (**a**,**b**,**c**), after treatment with a 17% EDTA (Germ EDTA 17%) (**d**,**e**,**f**), and after immersion in SBF for 24 h (**g**,**h**,**i**) and two months (**l**,**m**,**n**).

**Figure 4:**
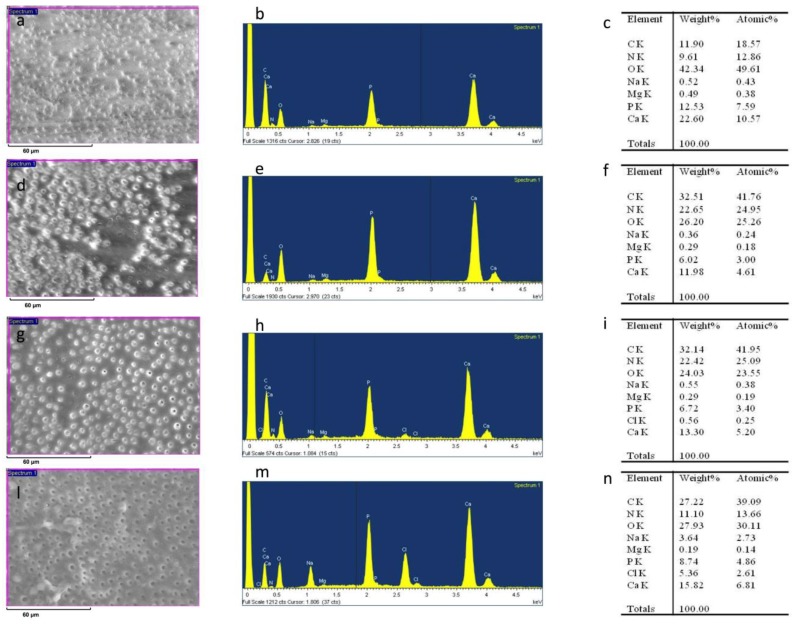
ESEM image, EDX spectra, and table of elements recorded on the dentin surface after sanding with abrasive paper (**a**,**b**,**c**), after treatment with a 10% citric acid solution (**d**,**e**,**f**), and after immersion in SBF for 24 h (**g**,**h**,**i**) and two months (**l**,**m**,**n**).

**Figure 5:**
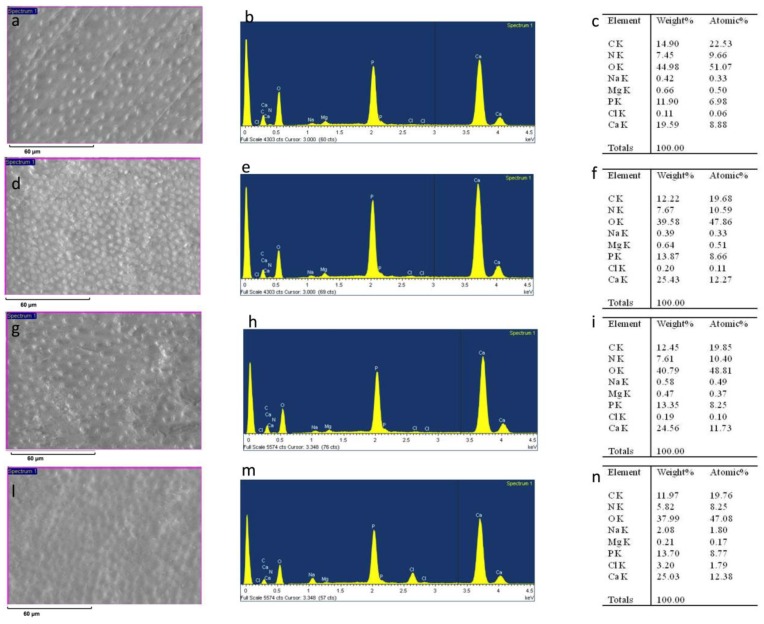
ESEM image, EDX spectra, and table of elements recorded on the dentin surface after sanding with abrasive paper (**a**,**b**,**c**), after washing with distilled water (**d**,**e**,**f**), and after immersion in SBF for 24 h (**g**,**h**,**i**) and two months (**l**,**m**,**n**).

**Figure 6:**
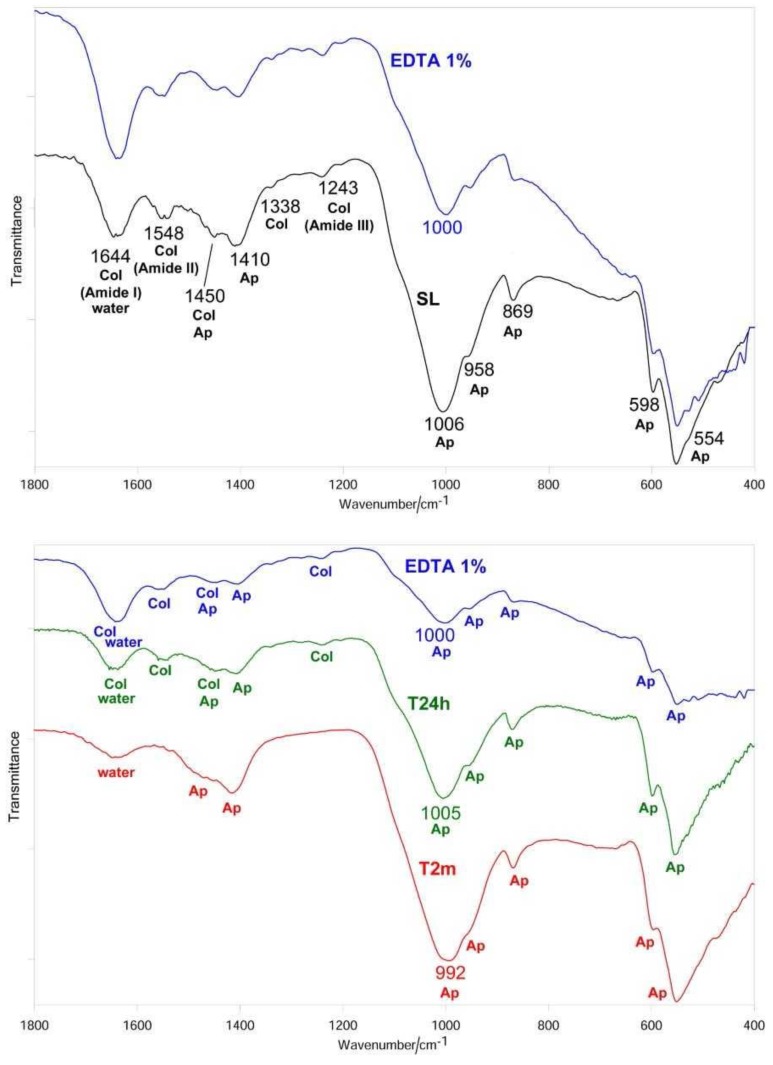
Average IR spectra recorded on the dentin surface after sanding (smear layer (SL), black), treatment with 1% EDTA (blue), aging in SBF for 24 h (T24h, green) and two months (T2m, red). The bands prevalently assignable to collagen (Col), water, and B-type carbonated apatite (Ap) are indicated.

**Figure 7:**
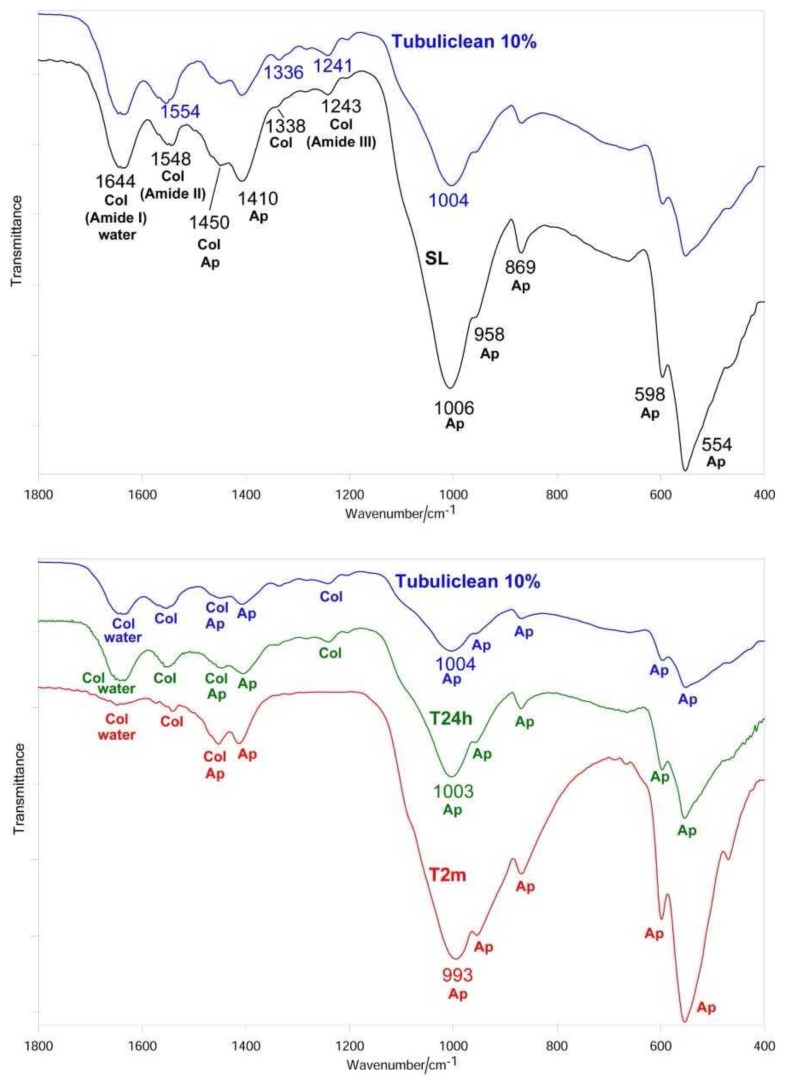
Average IR spectra recorded on the dentin surface after sanding (SL, black), treatment with 10% EDTA (Tubuliclean 10%) (blue), aging in SBF for 24 h (T24h, green) and two months (T2m, red). The bands prevalently assignable to collagen (Col), water, and B-type carbonated apatite (Ap) are indicated.

**Figure 8:**
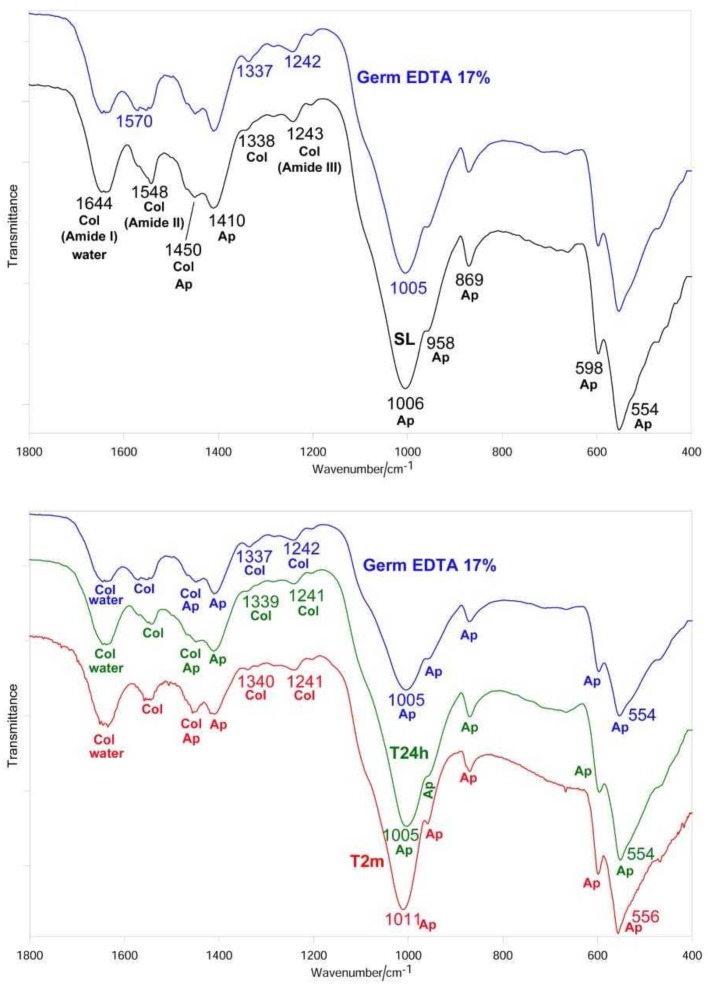
Average IR spectra recorded on the dentin surface after sanding (SL, black), treatment with 17% EDTA (Germ EDTA 17%) (blue), aging in SBF for 24 h (T24h, green) and two months (T2m, red). The bands prevalently assignable to collagen (Col), water, and B-type carbonated apatite (Ap) are indicated.

**Figure 9:**
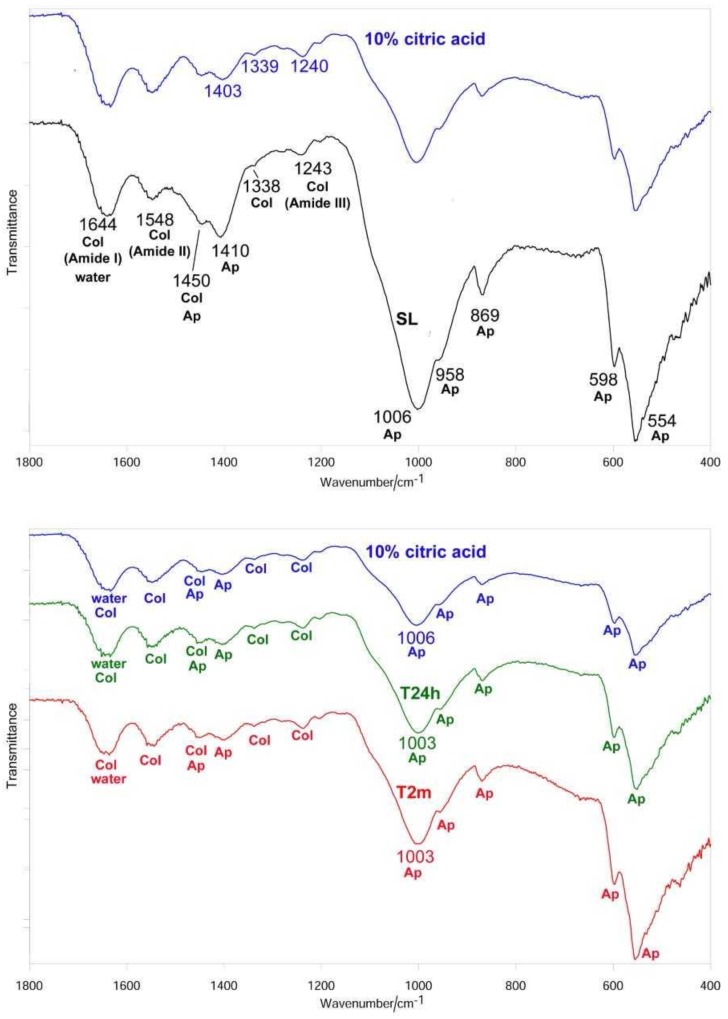
Average IR spectra recorded on the dentin surface after sanding (SL, black), treatment with 10% citric acid solution (blue), aging in SBF for 24 h (T24h, green) and two months (T2m, red). The bands prevalently assignable to collagen (Col), water, and B-type carbonated apatite (Ap) are indicated.

**Figure 10:**
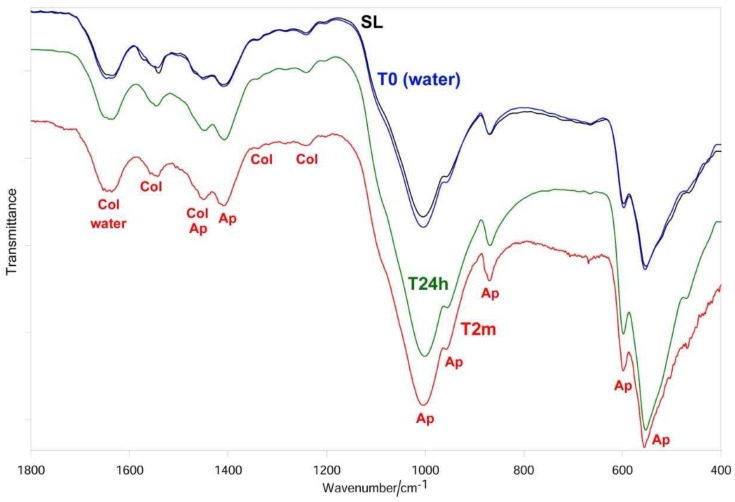
Average IR spectra recorded on the control dentin surface after sanding (SL, black), treatment with water (blue), aging in SBF for 24 h (T24h, green) and two months (T2m, red). The bands prevalently assignable to collagen (Col), water, and B-type carbonated apatite (Ap) are indicated.

After sanding the surface with abrasive paper, ESEM showed a uniform and homogeneous smear layer covering the tubules, see Figure 1a, Figure 2a, Figure 3a, Figure 4a, and Figure 5a, and high peaks of calcium (Ca), phosphorous (P), and oxygen (O) were detected by EDX analysis; also nitrogen (N) and carbon (C) were found, as shown in Figure 1b, Figure 2b, Figure 3b, Figure 4b, and Figure 5b. The Ca/N ratio ranged between 0.72 ± 0.05 and 1.09 ± 0.06, see Figure 1c, Figure 2c, Figure 3c, Figure 4c, Figure 5d, and Table 2; the P/N ratio was comprised between 0.53 ± 0.06 and 0.79 ± 0.03, see Figure 1c, Figure 2c, Figure 3c, Figure 4c, Figure 5d, and Table 3. The Ca/P ratio ranged between 1.27 ± 0.01 and 1.38 ± 0.03, see Figure 1c, Figure 2c, Figure 3c, Figure 4c, Figure 5d, and Table 4. The IR spectra of the SL samples showed bands typical of collagen and B-type carbonated apatite, see Figure 6, Figure 7, Figure 8, Figure 9 and Figure 10, at wavenumber values similar to those found for sound dentine [22,24].

To evaluate the relative apatite/collagen ratio, the IR *I*_apatite_/*I*_amide II_ ratio absorbance ratio was calculated, where *I*_apatite_ is the absorbance of the main apatite band (i.e., the ν_3_ PO_4_^3−^ mode at about 1000 cm^−1^ [31]), while *I*_amide II_ is the absorbance of the Amide II collagen band.

**Figure 11:**
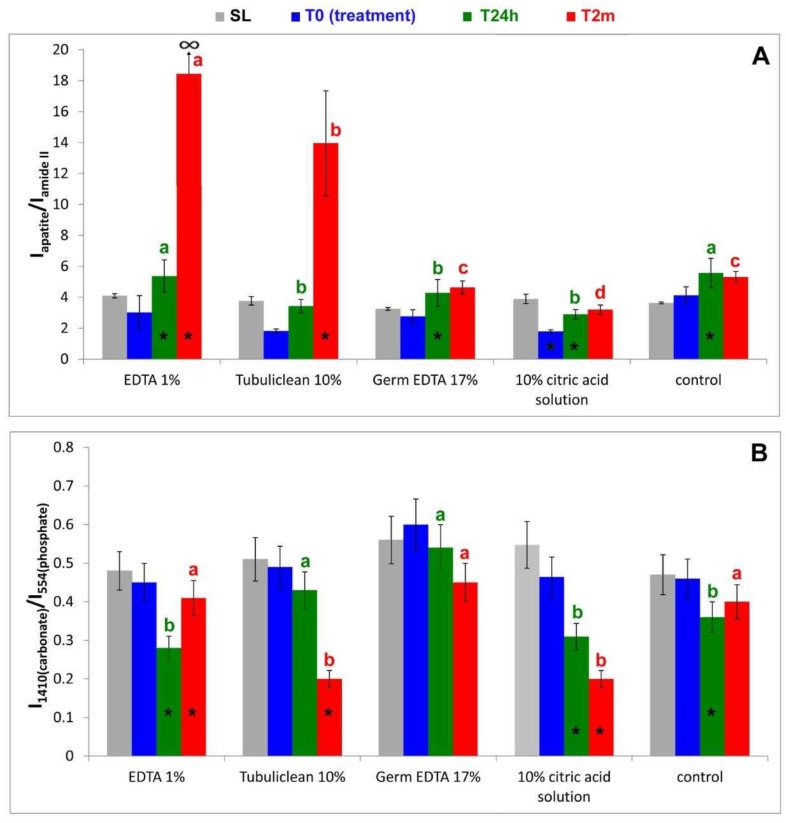
Values of the *I*apatite/*I*amide _II_ (**A**) and I1410(carbonate)/I554(phosphate) (**B**) ratios (average ± standard deviation), as calculated from the IR spectra recorded on the dentin surfaces under study, submitted to sanding (SL, grey), treatment with 1% EDTA, 10% EDTA (Tubuliclean 10%), 17% EDTA (Germ EDTA 17%), 10% citric acid solution, or water (blue), aging in SBF for 24 h (T24h, green) and two months (T2m, red). The asterisks on the histogram bars indicated significant differences with respect to the value observed in the previous step of the study within the same treatment. Identical letters represent no statistical differences (*P* ≥ 0.05) among tested solutions at T24h or T2m.

According to the literature [32,33], the carbonate content of the B-type carbonated apatite phase was assessed using the IR *I*_1410(carbonate)_/*I*_554(phosphate)_ ratio, where *I*_1410(carbonate)_ and *I*_554(phosphate)_ are the absorbances of the main carbonate band and ν_4_ PO_4_^3−^ mode, respectively. The values of these ratios are reported in Figure 11.

As expected, no significant differences in the *I*_apatite_/*I*_amide II_ and *I*_1410(carbonate)_/*I*_554(phosphate)_ ratios, see Figure 11, as well as in the Ca/N, P/N and Ca/P ratios, see Table 2, Table 3 and Table 4, were observed among the sanded samples (SL) corresponding to the different treatments.

### 3.1. 1% EDTA

After treatment with the chelating agent, ESEM showed that the dentinal tubules were completely open; smear plugs were absent, resulting in dentinal tubules that were completely free from debris, see Figure 1d. EDX showed high peaks of Ca and P, with similar intensities to those after sanding with abrasive paper; conversely, the O peak was weaker, while the signals from N and C were stronger, due to their increased contribution to dentine composition following demineralization, see Figure 1e.

The occurrence of this process was confirmed by the decrease of the Ca/N and P/N ratios upon the treatment; the former decreased from 0.84 ± 0.06 to 0.59 ± 0.05, see Figure 1f and Table 2, the latter from 0.62 ± 0.05 to 0.41 ± 0.02, see Figure 1f and Table 3. However, these changes were not statistically significant (*P* > 0.05). The Ca/P ratio significantly increased from 1.35 ± 0.01 to 1.43 ± 0.06, see Figure 1f and Table 4.

IR spectroscopy confirmed the occurrence of demineralization; in fact, the bands assigned to B-type carbonated apatite decreased in intensity with respect to those of collagen, without disappearing, see Figure 6. The Amide bands did not undergo any shift upon treatment; on the contrary, the ν_3_ PO_4_^3−^ band shifted from 1006 to 1000 cm^−1^. Wavenumber positions of the main IR bands of collagen and apatite are reported in Table 5.

The *I*_apatite_/*I*_amide II_ ratio decreased upon treatment with 1% EDTA, see Figure 11A, although its change was not significant (*P* > 0.05), in agreement with the EDX data. No significant variations in the *I*_1410(carbonate)_/*I*_554(phosphate)_ ratio were observed, see Figure 11B.

After immersion in SBF for 24 h, ESEM showed open dentinal tubules and small precipitates that are not able to occlude all the tubules, see Figure 1g.

EDX showed peaks of Ca and P similar to those after sanding and after treatment at T0. N and C levels were similar to those immediately after treatment. The chlorine (Cl) peak appeared upon immersion in SBF, see Figure 1h. Both the Ca/N and P/N ratios increased slightly, see Figure 1i and Table 2 and Table 3, but not significantly, suggesting only a small remineralization. The Ca/P ratio remained unchanged, see Figure 1i and Table 4. After aging in SBF for two months, ESEM showed several precipitates on the surface, see Figure 1l.

EDX showed high peaks of Ca, P, and O; high levels of N and C were also registered together with some Cl, see Figure 1m. The Ca/N and P/N ratios underwent significant increases, suggesting the occurrence of a marked degree of remineralization; at the same time, the Ca/P ratio resembled that observed after sanding, see Figure 1n and Table 2, Table 3 and Table 4.

IR spectroscopy confirmed the progressive remineralization observed by ESEM/EDX. Upon aging in SBF for 24 h, see Figure 6, the apatite bands were found to strengthen with respect to those assignable to collagen and the *I*_apatite_/*I*_amide II_ ratio significantly increased, see Figure 11A; after two months, the collagen amide II and III bands became undetectable, so that *I*_apatite_/*I*_amide II_ → ∞, see Figure 11A. At 24 h of aging, the ν_3_ PO_4_^3−^ band shifted to 1005 cm^−1^, i.e., to a wavenumber value similar to that observed upon sanding; a further shift to lower wavenumber values was observed after two months, see Table 5. With respect to the SL sample, the ν_3_ PO_4_^3−^ band fell at a lower wavenumber value and was visibly broadened, see Figure 6. 

At 24 h of aging, the *I*_1410(carbonate)_/*I*_554(phosphate)_ ratio (and thus carbonate content) was lower than in the SL sample, see Figure 11B; after two months, it increased attaining nearly the same value observed in the SL sample.

### 3.2. 10% EDTA

After treatment with the chelating agent, ESEM showed that the dentinal tubules were completely open and completely free from debris with some precipitates present on the surface, see Figure 2d. EDX still showed peaks of Ca, P, and O but with lower values than before. Conversely, as previously observed for the treatment with the 1% EDTA solution, N and C values were higher than before, see Figure 2e. The Ca/P ratio remained unchanged, see Figure 2f and Table 4. Both the Ca/N and P/N ratios decreased, see Figure 2f and Table 2 and Table 3, but only the change of the latter parameter appeared significant (from 0.61 ± 0.07 to 0.29 ± 0.02), suggesting the occurrence of a marked demineralization after the treatment.

IR spectroscopy confirmed this behavior, showing the already observed decrease in the relative intensity of the apatite bands, see Figure 7, and thus, in the *I*_apatite_/*I*_amide II_ ratio, see Figure 11A. The treatment with 10% EDTA induced a shift in the collagen Amide II and III bands, as well as in the COO^−^ stretching band at about 1338 cm^−1^, see Table 5; the shift in the ν_3_ PO_4_^3−^ band was lower than upon treatment with 1% EDTA, see Table 5. No significant variations in the *I*_1410(carbonate)_/*I*_554(phosphate)_ ratio were observed, as shown in Figure 11B.

After immersion in SBF for 24 h, ESEM showed few precipitates and all tubules were clearly open, see Figure 2g. EDX showed peaks that were very similar to those at T0, as shown in Figure 2h; the Ca/N, P/N and Ca/P ratios remained unchanged compared with T0, see Figure 2i and Table 2, Table 3 and Table 4, suggesting that the treatment with 10% EDTA had prevented remineralization at this stage. After immersion in SBF for two months, ESEM showed some dentinal tubules were still open, as shown in Figure 2l.

EDX showed intense peaks of Ca, P, and O; N and C levels were also high, see Figure 2m. The Ca/N and P/N ratios appeared increased at this stage, although the high standard deviation associated with the measurements meant the changes were not statistically significant, see Figure 2n and Table 2, Table 3 and Table 4. These trends showed that at this stage remineralization occurred.

The IR spectra confirmed these trends, see Figure 7. After 24 h of aging the *I*_apatite_/*I*_amide II_ ratio increased, but not significantly, while at two months the increase became significant, see Figure 11A; at this stage, the amide III band became undetectable, while the amide I and II bands were observed as weak spectral features, see Figure 7. With respect to the sanded sample, the ν_3_ PO_4_^3–^ band shifted to lower wavenumber value and broadened, see Figure 7 and Table 5. No significant variations in the *I*_1410(carbonate)_/*I*_554(phosphate)_ ratio were observed at 24 h, while after two months a significant decrease in the carbonate content of the B-type carbonated apatite was observed, as shown in Figure 11B.

### 3.3. 17% EDTA

After treatment with the chelating agent, ESEM showed that most of the dentinal tubules were completely open; smear plugs were absent. Peritubular dentine was compact and homogeneous, see Figure 3d. EDX showed the same peaks as before; the O level was a little lower, see Figure 3e. The Ca/N and P/N ratios remained practically unchanged upon treatment, while the Ca/P ratio increased, as previously observed for 1% EDTA, see Figure 3f and Table 2, Table 3 and Table 4. IR spectra showed only an insignificant reduction of the *I*_apatite_/*I*_amide II_ ratio, see Figure 11A, in agreement with the EDX results. The Amide II and III bands shifted upon treatment, as well as the COO^−^ stretching mode and the ν_3_ PO_4_^3−^ band, see Figure 8 and Table 5. No significant variations in the *I*_1410(carbonate)_/*I*_554(phosphate)_ ratio were observed, see Figure 11B.

After immersion in SBF for 24 h, ESEM showed open dentinal tubules and small precipitates that were not able to occlude all the tubules, see Figure 3g. EDX showed peaks and contents similar to those at T0, see Figure 3h, and no significant changes were observed in the Ca/N and P/N ratios, as shown in Figure 3i and Table 2 and Table 3, while the Ca/P ratio decreased, see Figure 3i and Table 4. After immersion in SBF for two months, ESEM showed a few precipitates and some open tubules, see Figure 3l.

EDX showed intense peaks of Ca, P, and O and high levels of C, see Figure 3m. Additionally, N and Cl were registered. As observed for the treatment with Tubuliclean 10%, at this stage, remineralization occurred, as indicated by the significant increases in the Ca/N and P/N ratios, see Figure 3n and Table 2 and Table 3; the former increased from 0.66 ± 0.06 to 1.56 ± 0.39, the latter from 0.47 ± 0.04 to 1.11 ± 0.27.

IR spectra, see Figure 8, showed that after 24 h of aging, the relative intensity of the apatite bands and thus the *I*_apatite_/*I*_amide II_ ratio, shown in Figure 11A, significantly increased; no further increases were observed after two months. The Amide III, the COO^−^ stretching mode, and the ν_3_ PO_4_^3−^ and ν_4_ PO_4_^3−^ bands underwent significant shifts in their wavenumber values, see Table 5. It is interesting to note that the ν_3_ PO_4_^3−^ mode shifted in an opposite manner with respect to the previous samples and sharpened rather than broadened. No significant variations in the *I*_1410(carbonate)_/*I*_554(phosphate)_ ratio were observed at 24 h and two months, see Figure 11B.

### 3.4. Citric Acid Solution

After treatment with the citric acid solution, ESEM showed that all dentinal tubules were completely open; smear plugs were absent, resulting in dentinal tubules that were completely free from debris. Peritubular dentine was compact and homogeneous, see Figure 4d.

EDX showed lower peaks of Ca, P, and O than before and higher levels of N and C, see Figure 4e. The Ca/N and P/N ratios significantly decreased upon treatment, suggesting the occurrence of a very strong demineralization, see Figure 4f and Table 2 and Table 3; the former decreased from 1.09 ± 0.06 to 0.26 ± 0.02 and the latter from 0.79 ± 0.03 to 0.19 ± 0.01. The Ca/P ratio decreased immediately after treatment and increased after 24 h and two months, see Figure 4f and Table 4. IR spectroscopy, see Figure 9, confirmed this result: the *I*_apatite_/*I*_amide II_ ratio, see Figure 11A, significantly decreased from 3.9 ± 0.3 to 1.8 ± 0.2 and certain shifts in the collagen Amide III and COO^−^ stretching bands were detected, see Table 5. No significant variations in the *I*_1410(carbonate)_/*I*_554(phosphate)_ ratio were observed, as shown in Figure 11B. After immersion in SBF for 24 h, ESEM showed open dentinal tubules and precipitates were absent, see Figure 4g. EDX showed component levels that were similar to those at T0, see Figure 4h, and accordingly, the Ca/N and P/N ratios did not undergo any significant change, see Figure 4i and Table 2, Table 3 and Table 4. After immersion in SBF for two months, ESEM did not show precipitates and all dentinal tubules were clearly open, as shown in Figure 4l. EDX showed high peaks of Ca, P, O, N, and C; Cl was still present, see Figure 4m. The Ca/N and P/N ratios remained constant; the Ca/P ratio decreased, see Figure 4n and Table 2, Table 3 and Table 4. The trend of the *I*_apatite_/*I*_amide II_ ratio, see Figure 11A, showed that after 24 h, a certain remineralization occurred, whilst no further apatite deposition was observed at two months.

### 3.5. Control

ESEM showed a uniform smear layer covering the tubules, see Figure 5d. After 24 h and two months the tubules were still closed.

EDX analysis showed high peaks of P, Ca, and O, that slightly decrease after 24 h and two months. After the removal of the smear layer with distilled water, Ca/N and P/N ratios increased (from 0.83 ± 0.08 to 1.17 ± 0.04 and from 0.65 ± 0.06 to 0.82 ± 0.03). After 24 h, both ratios slightly decreased, while increased after two months. The Ca/P ratio increased after washing and then remained constant.

As expected, the treatment with water (T0) did not alter the IR spectrum, as shown in Figure 10, and thus the *I*_apatite_/*I*_amide II_ and *I*_1410(carbonate)_/*I*_554(phosphate)_ ratios also remained the same, see Figure 11. Interestingly, immersion in SBF for 24 h induced a strengthening of the apatite bands as well as of the *I*_apatite_/*I*_amide II_ ratio, see Figure 11A, suggesting a certain remineralization; the carbonate content of the B-type carbonated apatite decreased, as revealed by the decrease of the *I*_1410(carbonate)_/*I*_554(phosphate)_ ratio, see Figure 11B. No further changes in the two ratios were observed after aging for two months.

## 4. Discussion

In this study, ATR/IR spectroscopy was used as a sensitive non-destructive analytical method for the assessment of the mineral content variations on the dentine surface and collagen modifications after the use of EDTA and citric acid. Several techniques, such as XRD or mass spectroscopy, require destructive processes (such as the pulverization of samples), which make the analysis of the solely treated area unreliable. Moreover, XRD can only provide information regarding dentine mineral components. Different studies have compared the chelating action and effectiveness of citric acid solutions vs. EDTA solutions in removing the smear layer when used as root canal irrigants [34,35,36,37,38]. Two studies have analyzed organic and inorganic dentine tissues’ modifications by FTIR [39,40], proving the suitability of this technique for the qualitative and semiquantitative analysis of biological samples. One study has analyzed the chemical degradation of ground dentine particles constituents (namely apatite and collagen) before and after irrigation with 2.5% NaOCl and/or 17% EDTA using the FTIR technique [39], revealing a continuing apatite depletion (loss of phosphates) over a period of 24 h when only EDTA solution is used. This data is similar to the result obtained in the present study. Another study has investigated the demineralization effect of several concentrations of EDTA alone or in combination with NaOCl on bovine dentine slices using ATR-FTIR [40]. This study has reported a greater dentine demineralization at higher concentrations and time of treatment. However, both studies did not investigate dentine mineralization changes over longer times (i.e., two months). Again, the activity of residual chelating agents could significantly affect the tooth microstructure.

The IR spectra of all the treated dentine samples at T0 showed the weakening of the apatite bands, see Figure 6, Figure 7, Figure 8 and Figure 9; however, it must be observed that the apatite bands did not disappear. This behavior suggests that demineralization was only partial and took place at a lower depth than that corresponding to the ATR sampling (i.e., 2 µm), differing from our previous studies in which dentine samples were treated with 17% EDTA for 2 h [24]. This trend confirms that the application time of the solution is crucial in determining the decalcifying effect [15].

The *I*_apatite_/*I*_amide II_ ratio was found to decrease upon all the treatments, see Figure 11A; the treatment with 10% citric acid solution and 10% EDTA determined the highest % decreases in this parameter and thus the most pronounced demineralization within the uppermost 2 µm region investigated by ATR. The Ca/N and P/N ratios were also found to decrease upon all the treatments; however, only 10% EDTA and 10% citric acid solution induced significant changes in these parameters, in agreement with the IR findings.

The carbonate content of the B-type carbonated apatite was evaluated through the IR *I*_1410(carbonate)_/*I*_554(phosphate)_ ratio; it did not change significantly, see Figure 11B, upon all the treatments. This behavior suggests that the chelating agents removed apatite without altering the carbonate content of the phase that remained in the partially demineralized dentine sample. This result is in agreement with the recent findings on dentine slices treated for 1 min with differently concentrated EDTA solutions [28].

All the treatments, with the exception of 1% EDTA, determined shifts in the collagen bands (i.e., Amide III, COO^−^ modes and, in the case of 10% and 17% EDTA, also Amide II, see Figure 7, Figure 8 and Figure 9). The Amide bands, due to the repeating peptide unit of proteins, have been extensively used to study the secondary structure and conformational transitions of proteins; the observed changes suggest that the collagen network underwent a certain conformational rearrangement upon demineralization, although to a lower extent than that previously observed by treatment with 17% EDTA for a longer period (i.e., 2 h [24]). From this point of view, it is not surprising that the treatment with 1% EDTA (i.e., the lowest investigated concentration) did not induce any effect on collagen conformation, see Figure 6. The shifts of the COO^−^ collagen vibration may be ascribed to changes in the interactions between the collagen and apatite phases, according to the literature [41].

The concept of remineralization is based on the reincorporation of mineral (apatite) in dental tissues (dentine or enamel [24]). In the present study, looking at remineralization represents additional proof of the action of the chelating agents during the evaluation times. After 24 h of aging in SBF, IR spectroscopy showed that the *I*_apatite_/*I*_amide II_ increased, and thus remineralization occurred for all the samples, control included. Moreover, it must be observed that all the T24h samples were characterized by *I*_apatite_/*I*_amide II_ ratios which were significantly different (i.e., lower) with respect to the control sample, except that corresponding to the treatment with 1% EDTA, which had an *I*_apatite_/*I*_amide II_ comparable to the control sample, see Figure 11A. This result suggests that the treatment with 1% EDTA did not prevent remineralization and this sample behaved similarly to the control one, which, upon aging in SBF for 24 h, remineralized to a certain extent. On the contrary, treatment with the other chelating agents hindered remineralization at this stage.

The IR analysis of the T24h sample previously treated with 1% EDTA showed that the deposited inorganic phase was less crystalline than that present in the SL sample, as revealed by the higher width of the ν_3_ PO_4_^3−^ band, see Figure 6; moreover, as suggested by the *I*_1410(carbonate)_/*I*_554(phosphate)_ value, see Figure 11B, it had a lower carbonate content, in agreement with the literature [25]. Analogous trends were observed for the control sample, see Figure 10 and Figure 11B. On the basis of these results, it may be affirmed that the newly formed mineral layer on the surface of dentine is different from that of the smear layer.

After two months of aging, the *I*_apatite_/*I*_amide II_ ratio and thus the degree of remineralization significantly increased for all the samples, except those treated with 10% citric acid solution and 17% EDTA; in fact, all the samples except the latter two had *I*_apatite_/*I*_amide II_ ratios higher than the control sample that decreased along the series EDTA 1% > 10% EDTA > 17% EDTA > 10% citric acid. It is interesting to note that the IR spectrum of the T2m sample previously treated with 1% EDTA did not show the Amide II and III bands, see Figure 6, suggesting that the deposited apatite phase was thicker than the sampling depth analyzed by ATR (i.e., 2 µm). Therefore, the obtained IR spectrum, shown in Figure 6, is mainly representative of the phase formed upon aging and allows information to be gained on its crystallinity and carbonate content; the width of the previously mentioned ν_3_ PO_4_^3−^ band (which appeared at a wavenumber shifted to 992 cm^−1^) was higher than for the SL sample, suggesting that the inorganic phase was less crystalline; however, as suggested by the *I*_1410(carbonate)_/*I*_554(phosphate)_ value, shown in Figure 11B, it had a carbonate content similar to that present in the smear layer. The phase formed on the dentine sample previously treated with 10% EDTA after two months was even less crystalline, see Figure 7, and contained a significantly lower amount of carbonate, as shown in Figure 11B. Upon treatment with citric acid, the mineral phase formed after two months was characterized by nearly the same carbonate content as the latter sample, see Figure 11B, but was more crystalline, see Figure 9; however, it must be observed that for this sample the spectral contribution of the underlying dentine is higher, due to the stronger relative intensity of its bands.

On the basis of the IR analyses, the remineralization extent was found to decrease along the series; EDTA 1% > 10% EDTA > 17% EDTA > 10% citric acid. The ESEM/EDX data did not show a perfectly coincident trend, probably due to the different sampling areas of the two techniques. However, ESEM/EDX and IR spectroscopy were in agreement that the highest degree of remineralization was promoted by 1% EDTA and the lowest degree of remineralization was found after treatment with 10% citric acid.

Based on these findings, remineralization was most greatly observed on samples treated with low concentrations (in particular, samples treated with 1% EDTA), also showing lower collagen rearrangement. Differently, more concentrated solutions demonstrated a higher demineralization effect and higher collagen rearrangements. Demineralization and collagen rearrangement may have potential negative effects on root dentine and may play a negative effect on tooth longevity [42].

This effect may potentially have implications on the outcome of endodontic treatments and, therefore, the use of these solutions should be reconsidered. Further studies with more samples need to be performed to validate these results.

## 5. Conclusions

Key findings of this article may be summarized as follows:

- *I*_apatite_/*I*_amide II_ and Ca/N and P/N ratios were reduced after 10 min of treatment with chelating agents, suggesting a demineralization of dentine (higher demineralization in 10% citric acid and lower in 17% EDTA).
- No significant changes in *I*_1410(carbonate)_/*I*_554(phosphate)_ ratio were observed after 10 min; chelating agents removed only apatite and not carbonate in the demineralized samples.
- Shift in collagen IR Amide II and III bands after treatment with 10% EDTA, 17% EDTA, and 10% citric acid; the collagen network performed a rearrangement.
- *I*_apatite_/*I*_amide II_ ratio increased after 24 h in SBF; a remineralization occurred in each sample; those treated with 1% EDTA had a similar ratio to the control group.
- *I*_1410(carbonate)_/*I*_554(phosphate)_ ratio decreased in 1% EDTA treated samples after 24 h; there was a reduction in carbonate content.
- *I*_apatite_/*I*_amide II_ ratio increased after two months from the treatment with 1% and 10% EDTA; Ca/N and P/N ratios, in addition to increasing in previously mentioned treatments, also increased after treatment with 17% EDTA. Results indicate dentine remineralization in all cases.
- Both ESEM/EDX and IR spectroscopy techniques revealed that treatment with 1% EDTA yielded the highest remineralization, while treatment with 10% citric acid yielded the lowest.

## Figures and Tables

**Table 1:** Tested chelating agents.

Treatments	Manufacturing	Components
1% EDTA	Experimental solution obtained by dilution of Tubuliclean 10% with distilled water	1% ethylenediaminetetraacetic acid in deionized water, pH 7.4
10% EDTA (Tubuliclean 10%)	OGNA (Muggiò, MI, Italy)	10% ethylenediaminetetraacetic acid (EDTA) buffered at neutral pH, pH 6.8
17% EDTA (Germ EDTA 17%)	GermDental (Kerr, Scafati, SA, Itay)	17% ethylenediaminetetraacetic acid (EDTA), pH 9.9
10% Citric acid	Experimental solution obtained by the solubilization of 99% citric acid monohydrate (Sigma Aldrich, Saint Louis, MO, USA) in distilled water	10% citric acid monohydrate in deionized water, pH 1.8

**Table 2:** Ca/N atomic ratios (mean ± standard deviation (SD)). In columns, identical superscript lowercase letters represent no statistical differences (two-way analysis of variance (ANOVA) followed by an RM Student–Newman–Keuls test) among tested solutions (*P* ≥ 0.05). In the horizontal rows, identical superscript uppercase letters represent no statistical differences (one-way ANOVA for repeated measures followed by an RM Student–Newman–Keuls test) among evaluation times (*P* ≥ 0.05).

Treatments	Smear Layer	T0	24 Hours	2 Months
1% EDTA	0.84 ± 0.06 ^Aa^	0.59 ± 0.05 ^Aa^	0.67 ± 0.14 ^Aa^	2.34 ± 0.51 ^Ba^
10% EDTA	0.85 ± 0.09 ^Aa^	0.40 ± 0.03 ^Ba^	0.31 ± 0.03 ^Bb^	0.91 ± 0.49 ^Ab^
17% EDTA	0.72 ± 0.05 ^Ab^	0.70 ± 0.04 ^Ab^	0.66 ± 0.06 ^Aa^	1.56 ± 0.39 ^Bc^
10% Citric acid	1.09 ± 0.06 ^Ac^	0.26 ± 0.02 ^Bc^	0.31 ± 0.03 ^Bb^	0.33 ± 0.01 ^Bd^
Control	0.83 ± 0.08 ^Aa^	1.17 ± 0.04 ^Bd^	1.12 ± 0.08 ^Bc^	1.45 ± 0.19 ^Bc^

**Table 3:** P/N atomic ratios (mean ± SD). In columns, identical superscript lowercase letters represent no statistical differences (two-way ANOVA followed by an RM Student–Newman–Keuls test) among tested solutions (*P* ≥ 0.05). In the horizontal rows, identical superscript uppercase letters represent no statistical differences (one-way ANOVA for repeated measures followed by an RM Student–Newman–Keuls test) among evaluation times (*P* ≥ 0.05).

Treatments	Smear Layer	T0	24 Hours	2 Months
1% EDTA	0.62 ± 0.05 ^Aa^	0.41 ± 0.02 ^Aa^	0.48 ± 0.10 ^Aa^	1.80 ± 0.46 ^Ba^
10% EDTA	0.61 ± 0.07 ^Aa^	0.29 ± 0.02 ^Ba^	0.23 ± 0.03 ^Ba^	0.65 ± 0.35 ^Ab^
17% EDTA	0.53 ± 0.06 ^Aa^	0.48 ± 0.03 ^Aa^	0.47 ± 0.04 ^Aa^	1.11 ± 0.27 ^Bc^
10% Citric acid	0.79 ± 0.03 ^Aa^	0.19 ± 0.01 ^Ba^	0.22 ± 0.02 ^Ba^	0.22 ± 0.01 ^Bd^
Control	0.65 ± 0.06 ^Aa^	0.82 ± 0.03 ^Bb^	0.78 ± 0.06 ^Bb^	1.04± 0.14 ^Bc^

**Table 4:** Ca/P atomic ratios (mean ± SD). In columns, identical superscript lowercase letters represent no statistical differences (two-way ANOVA followed by an RM Student–Newman–Keuls test) among tested solutions (*P* ≥ 0.05). In the horizontal rows, identical superscript uppercase letters represent no statistical differences (one-way ANOVA for repeated measures followed by an RM Student–Newman–Keuls test) among evaluation times (*P* ≥ 0.05).

Treatments	Smear Layer	T0	24 Hours	2 Months
1% EDTA	1.35 ± 0.01 ^Aa^	1.43 ± 0.06 ^Ba^	1.41 ± 0.02 ^Ba^	1.31 ± 0.04 ^Aa^
10% EDTA	1.38 ± 0.01 ^Aa^	1.41 ± 0.04 ^Aa^	1.36 ± 0.01 ^Bb^	1.41 ± 0.01 ^Ab^
17% EDTA	1.32 ± 0.02 ^Ab^	1.47 ± 0.01 ^Bb^	1.40 ± 0.02 ^Ca^	1.40 ± 0.03 ^Cb^
10% Citric acid	1.38 ± 0.03 ^Aa^	1.33 ± 0.01 ^Bc^	1.43 ± 0.02 ^Ca^	1.45 ± 0.02 ^Cc^
Control	1.27 ± 0.01 ^Ac^	1.42 ± 0.00 ^Ba^	1.43 ± 0.01 ^Ba^	1.40 ± 0.01 ^Bb^

**Table 5:** IR band wavenumber positions (cm^−1^).

Treatments–Time	Collagen Amide II	Collagen COO^−^ Stretching Band	Collagen Amide III	Apatite, ν_3_ PO_4_^3^^−^ Stretching
Smear layer	1548	1338	1243	1006
1% EDTA–T0	1548	1338	1243	1000
24 hours	1548	1338	1243	1005
2 months	not detected	not detected	not detected	992
10% EDTA–T0	1554	1336	1241	1004
24 hours	1554	1336	1241	1003
2 months	very weak	not detected	not detected	993
17% EDTA–T0	1570–1548	1337	1242	1005
24 hours	1548	1339	1241	1005
2 months	1548	1340	1241	1011
10% citric acid–T0	1548	1339	1240	1006
24 hours	1548	1339	1240	1003
2 months	1548	1339	1240	1003
control–T0	1548	1338	1243	1006
24 hours	1548	1338	1243	1006
2 months	1548	1338	1243	1006
